# Surgical Outcomes of Intermittent Exotropia in Adults: A Systematic Review

**DOI:** 10.7759/cureus.102879

**Published:** 2026-02-03

**Authors:** Shie Wei Chan, Chi Kit Yan

**Affiliations:** 1 Ophthalmology, Manchester Royal Eye Hospital, Manchester, GBR; 2 Paediatric Ophthalmology, Manchester Royal Eye Hospital, Manchester, GBR

**Keywords:** a systematic review, exotropia, intermittent exotropia, strabismus surgery, surgical outcome

## Abstract

Adult intermittent exotropia (IXT) is less well studied than paediatric IXT, yet counselling is challenging due to postoperative exodrift, diplopia risk, and differing functional expectations. This systematic review synthesised surgical outcomes for adult (≥18 years) IXT. A Preferred Reporting Items for Systematic Reviews and Meta-Analyses (PRISMA) 2020-guided systematic review of PubMed/MEDLINE was performed using two searches (“surgical” AND “outcomes” AND “exotropia”; “surgery” AND “intermittent exotropia”), limited to English-language human studies published within the last 10 years. Eligible studies reported postoperative motor alignment and/or reoperation, diplopia, drift, or binocular outcomes in adults with IXT. Motor success was narratively standardised to ≤10 prism dioptres (PD) of orthotropia at distance (allowing ≤5 PD esodeviation where applicable). Risk of bias was assessed using the Risk Of Bias In Non-randomized Studies of Interventions (ROBINS-I). Due to heterogeneity in outcomes and follow-up, meta-analysis was not undertaken. Six studies (one randomised prospective interventional study and five retrospective cohorts) comprising 552 participants were included. Mean age ranged from 27 to 42 years, and preoperative distance deviation was typically 30-40 PD (one study >50 PD). Reported motor success ranged from 47.6% to 90% across primary endpoints of three months to two years or longer, with most studies demonstrating progressive exotropic drift over time. Diplopia was generally transient and varied by early postoperative alignment and intervention. No major adverse events occurred, and a low reoperation rate was reported. Overall risk of bias was serious in most studies, primarily due to confounding and selection bias. Adult IXT surgery achieves moderate-to-high motor success, but outcomes vary with technique, early alignment, and follow-up duration. Prospective adult-only studies with standardised endpoints and patient-centred outcomes are needed.

## Introduction and background

Pathophysiology

Intermittent exotropia (IXT) is one of the most common forms of childhood-onset strabismus and frequently persists into adulthood [1,2]. While the natural history and surgical management of IXT have been extensively studied in paediatric populations, far less attention has been given to outcomes in adults. This gap is clinically important, as adults with IXT differ substantially from children in terms of symptom burden, binocular adaptation, expectations of surgery, and tolerance of postoperative diplopia.

Symptoms

In adults, IXT may present with asthenopia, diplopia, reduced stereoacuity (3D vision), impaired binocular control, and psychosocial concerns [3,4]. Although many adult cases represent persistence of childhood-onset IXT, others may demonstrate progression in angle, frequency, or symptomatology over time. Surgical correction is often pursued to improve ocular alignment, alleviate symptoms, and restore binocular function. However, counselling adults regarding likely surgical outcomes is challenging, particularly with respect to long-term alignment stability, exodrift, and the risk of persistent or new-onset diplopia [5,6].

The adult versus paediatric gap

Most of the evidence guiding surgical decision-making in IXT is derived from paediatric cohorts or mixed-age studies, in which outcomes in adults are not reported separately. Extrapolating paediatric data to adult patients may be inappropriate, as sensory plasticity, the ability to fuse images, and postoperative adaptation differ significantly between age groups [7,8]. Adults may have a lower capacity for suppression, a higher risk of symptomatic double vision after overcorrection, and different patterns of postoperative drift. Consequently, the optimal surgical target, expected success rates, and risk profiles in adults remain uncertain.

Several adult-focused studies have explored surgical outcomes in IXT, including the impact of early postoperative alignment, degree of overcorrection or undercorrection, choice of surgical technique, and use of adjustable sutures [6,9,10]. These studies suggest that early postoperative alignment may influence long-term success and deviation of the alignment, but findings are heterogeneous, and reported success rates vary widely. Furthermore, most available studies are retrospective, non-randomised, and differ in definitions of success, follow-up duration, and outcome assessment, making synthesis of the evidence difficult [9,11].

Study objectives

To the best of our knowledge, no systematic review to date has specifically synthesised surgical outcomes of IXT in adults using contemporary methodological standards [12]. A focused appraisal of adult-only data is necessary to provide realistic expectations for patients, inform surgical planning, and identify areas where evidence remains limited.

The aim of this systematic review is to evaluate surgical outcomes of IXT in adults, including postoperative motor alignment success, reoperation rates, exodrift over time, and functional outcomes such as diplopia and binocular vision. By restricting inclusion to adult populations and critically appraising study quality using established risk-of-bias frameworks, this review seeks to provide clinically relevant guidance for ophthalmologists managing IXT in adult patients.

## Review

Methods

Study Design and Reporting Standards

**Figure 1:**
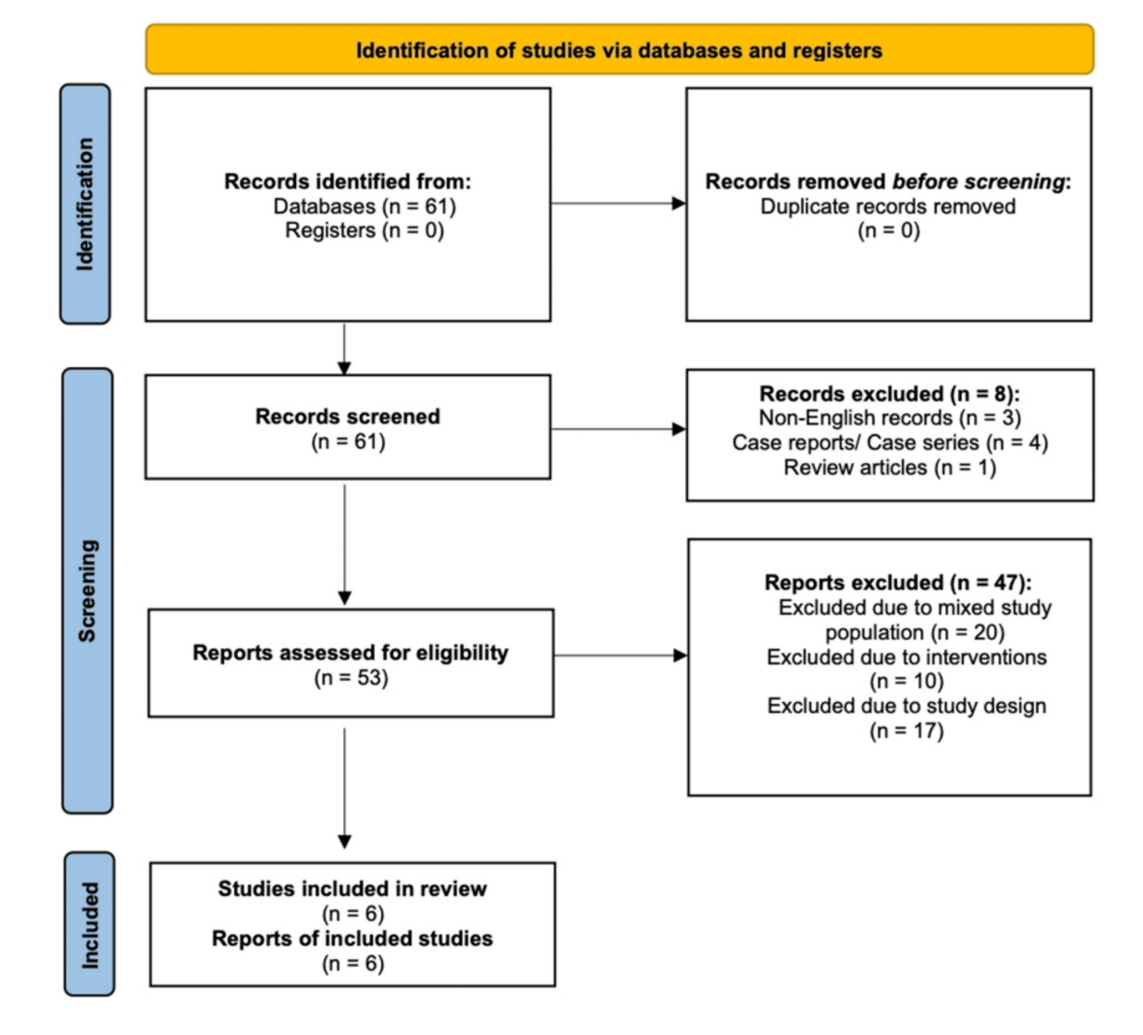
PRISMA flowchart representing the study selection process. PRISMA: Preferred Reporting Items for Systematic Reviews and Meta-Analyses

**Figure 2:**
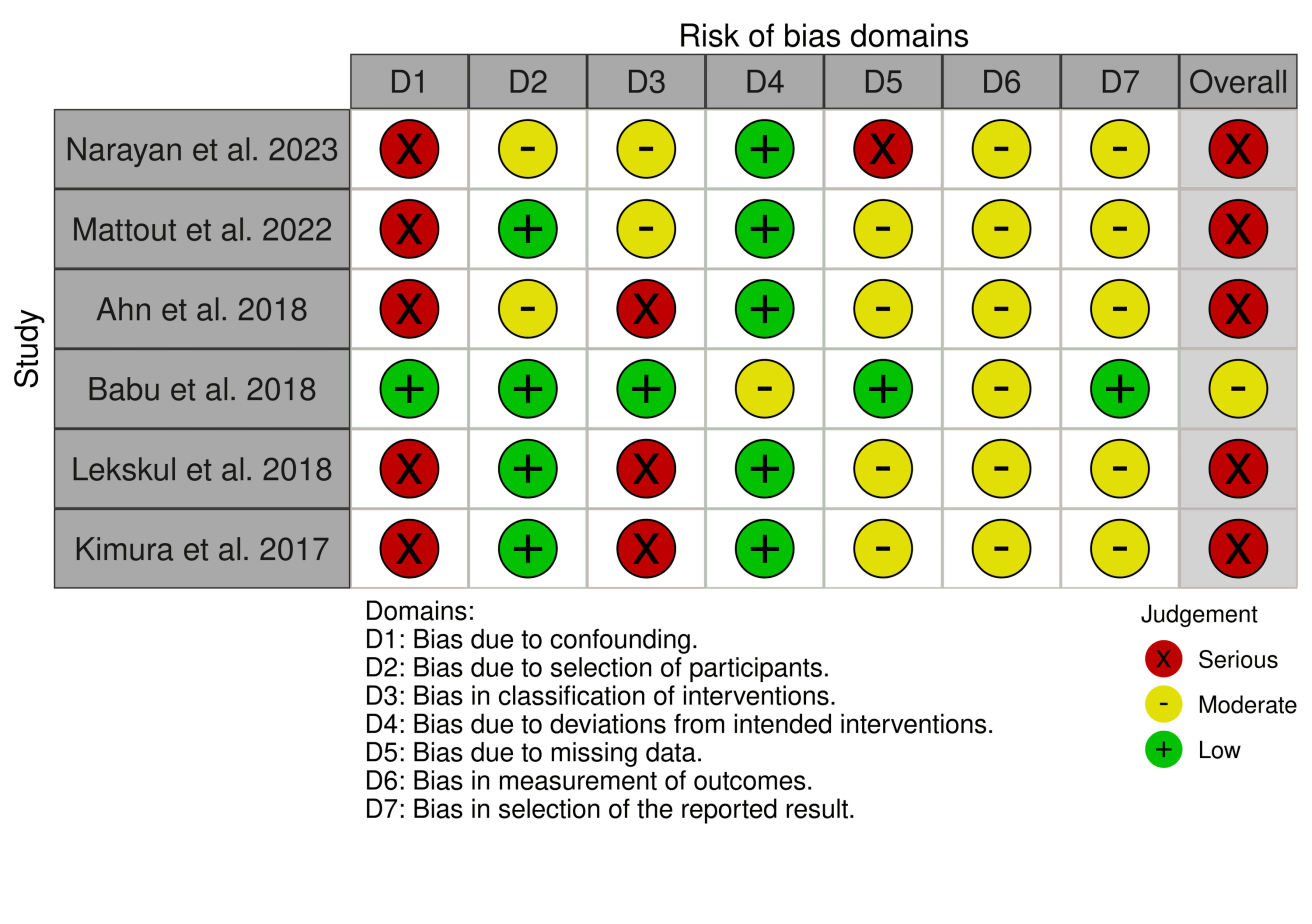
Visualization of the ROBINS-I assessment using a traffic light system, generated with the Robvis tool. ROBIN-I: Risk Of Bias In Non-randomised Studies of Interventions Studies included [6,9-11,14,15]

This systematic review was conducted in accordance with the Preferred Reporting Items for Systematic Reviews and Meta-Analyses (PRISMA) 2020 guidelines [12]. The study selection process is illustrated in Figure 1. Given the predominance of non-randomised surgical studies, risk of bias was assessed using the Risk Of Bias In Non-randomised Studies of Interventions (ROBINS-I) tool [13], with results summarised in Figure 2.

Eligibility Criteria

Studies were selected according to predefined eligibility criteria based on population, intervention, outcomes, and study design. Eligible studies included adults aged 18 years or older with a diagnosis of IXT who underwent surgical correction. Studies that enrolled mixed-age populations were included only if outcomes for adult patients were reported separately or were extractable. Surgical interventions included any strabismus surgery performed for IXT, such as bilateral lateral rectus recession, unilateral recess-resect procedures, adjustable or non-adjustable techniques, and muscle plication or resection. To be eligible, studies were required to report at least one relevant postoperative outcome, including motor alignment success, reoperation or recurrence, postoperative diplopia, exodrift, or sensory outcomes such as stereoacuity or fusion. Randomised controlled trials, prospective cohorts, and retrospective observational studies were included. Case reports, small case series (fewer than 10 patients), review articles, conference abstracts, basic science studies, and studies evaluating non-surgical interventions were excluded. Only articles published in English within the preceding 10 years were considered. Although definitions of surgical success varied slightly across studies, postoperative motor alignment was narratively standardised to alignment within ≤10 prism dioptres (PD) of orthotropia at distance, allowing for small esodeviations (≤5 PD) where applicable.

Information Sources and Search Strategy

A systematic literature search was conducted using the PubMed/MEDLINE database. Two complementary searches were performed to maximise sensitivity. The searches combined the terms “surgical,” “outcomes,” and “exotropia,” while the second search used the terms “surgery” and “intermittent exotropia.” Both searches incorporated free-text terms and Medical Subject Headings related to adult populations and strabismus surgery. Results were limited to human studies published in English within the preceding 10 years. Titles and abstracts were screened, and full texts were reviewed for eligibility. Duplicate records across searches were identified and removed. Reference lists of included studies were manually reviewed to identify additional eligible articles.

Study Selection

All records identified through the database search were screened at the title and abstract level. Full texts were retrieved for potentially eligible studies and assessed independently against the inclusion and exclusion criteria. Reasons for exclusion at the full-text stage were documented and categorised. The study selection process is summarised in Figure 1. After screening and full-text assessment, six studies met the inclusion criteria and were included in the final qualitative synthesis.

Data Extraction

Data were extracted from included studies using a standardised approach. Extracted information included study characteristics (year of publication, country, and study design), patient demographics and sample size, type of surgical intervention and alignment targets, duration of follow-up, and definitions of surgical success. Outcome data included postoperative motor alignment, reoperation or recurrence rates, exodrift over time, postoperative diplopia, sensory outcomes, and reported complications.

Data Synthesis

Due to substantial clinical and methodological heterogeneity across studies, including differences in surgical techniques, outcome definitions, and follow-up duration, quantitative meta-analysis was not appropriate, and results were synthesised narratively.

Risk of Bias Assessment

The risk of bias for each included study was assessed using the ROBINS-I tool. This tool evaluates bias across seven domains, including bias due to confounding, selection of participants, classification of interventions, deviations from intended interventions, missing data, measurement of outcomes, and selection of the reported result. Each domain was judged as having low, moderate, or serious risk of bias, and an overall risk of bias judgement was assigned according to the highest-risk domain.

Results

Risk of Bias Assessment

Given the predominance of retrospective, non-randomised surgical studies, most included studies were judged to have a serious overall risk of bias, primarily driven by confounding and selection bias. Particular attention was paid to sources of selection bias, loss to follow-up, and confounding by surgical indication, which were common across retrospective cohort designs. A summary of the risk of bias assessment is presented in Figure 2.

Six studies were included in this review, which consisted of one randomised prospective interventional study and five retrospective studies (two comparative cohorts and three retrospective cohorts). A total of 552 participants were included. The number of patients in each study ranged from 40 to 234. Mean age across the studies ranged from approximately 27 to 42 years. The preoperative distance deviation was commonly between 30 and 40 PD, with one study focusing on large angles >50 PD. Follow-up duration varied substantially from three months to two years or longer, with one large cohort reporting a mean follow-up of 43 months.

Follow-up completeness was variably reported. Kimura et al. excluded 21 patients lost to follow-up within 12 months [11]. Babu et al. reported no loss to follow-up [14]. Ahn et al. excluded participants with >10 PD exodeviation at postoperative week 1, and both alignment-group prognostic cohorts were susceptible to selection and follow-up bias inherent to retrospective designs [6,9]. Given heterogeneity in outcome definitions and primary endpoints, meta-analysis was not performed. The study characteristics are summarised in Table 1.

**Table 1:** Study characteristics. PD: prism dioptres; PR: plication-recession; R&R: LR recession and MR resection; AS: adjustable suture; NAS: non-adjustable suture; BTA-BLR: botulinum toxin augmented bilateral LR recession; BLR & uMR: bilateral LR recession and unilateral MR resection

Study number	Study author (year)	Design	Patients (n)	Mean age (years)	Preoperative distance deviation (PD)	Follow-up period
1	Ahn et al., 2018 [6]	Retrospective cohort	46	29.6 ± 11.6	33.4 ± 11.0	28.8 ± 6.1 months (≥2 years)
2	Lekskul et al., 2018 [10]	Retrospective cohort	234	27.56 (18-42)	Majority ≤30 (15-45)	Mean 42.84 months (6-87)
3	Kimura et al., 2017 [11]	Retrospective comparative cohort	88 (PR 45; R&R 43)	PR: 42.0 ± 22.9; R&R: 34.7 ± 25.3	PR 40.1 ± 12.9; R&R 40.0 ± 14.9	PR 21.0 ± 7.6 months; R&R 24.0 ± 8.6 months
4	Babu et al., 2017 [14]	Randomised prospective interventional	40 (AS 20; NAS 20)	AS 24.5 ± 4.12; NAS 24.5 ± 4.45	AS 35.40 ± 3.68; NAS 36.15 ± 4.03	6 months
5	Mattout et al., 2022 [15]	Retrospective comparative cohort	51 (BTA-BLR 21; BLR & uMR 30)	BTA-BLR 27.5 ± 5.86; BLR & uMR 27.7 ± 5.31	BTA-BLR 71.9 ± 6.7; BLR & uMR 72.7 ± 7.7	14.6 ± 4.7 months
6	Narayan et al., 2023 [9]	Retrospective cohort	93	37.4 ± 20.2	27 ± 12 (15-50)	264 ± 141 days (93-433)

Surgical Interventions

Three studies compared specific surgical approaches: plication-recession (PR) vs. LR recession and MR resection (R&R), adjustable sutures (AS) vs. non-adjustable sutures (NAS), and botulinum toxin augmented bilateral LR recession (BTA-BLR) vs. bilateral LR recession and unilateral MR resection (BLR & uMR) for large-angle IXT [11,14,15]. Two studies assessed early postoperative alignments as a prognostic factor rather than comparing surgical techniques [6,9]. Ahn et al. used mixed surgical procedures (BLR, unilateral LR recession, R&R), whereas Narayan et al. used R&R exclusively [6,9]. One large cohort used a consistent strategy of 5 PD undercorrection following R&R (Table 2) [10].

**Table 2:** Surgical interventions. BLR: bilateral LR recession; uLR: unilateral LR recession; R&R: LR recession and MR resection; PD: prism dioptress; PR: plication-recession; AS: adjustable suture; NAS: non-adjustable suture; BLR: bilateral LR recession; BTA-BLR: botulinum toxin augmented bilateral LR recession; BLR & uMR: bilateral LR recession and unilateral MR resection Grouping was performed based on early alignment.

Study	Intervention	Comparator	Key study detail
Ahn et al., 2018 [6]	Mixed procedures: BLR, uLR, R&R	None (alignment-group analysis)	Grouped by one-week postoperative alignment (Group A: any esotropia; Group B: orthophoria to ≤10 PD exotropia)
Lekskul et al., 2018 [10]	R&R	None	Intended 5 PD undercorrection
Kimura et al., 2017 [11]	PR	R&R	Compared PR vs R&R
Babu et al., 2018 [14]	AS BLR	NAS BLR	Randomised comparison of AS BLR vs NAS BLR
Mattout et al., 2022 [15]	BTA-BLR	BLR & uMR	Compared BTA-BLR vs BLR & uMR
Narayan et al., 2023 [9]	R&R	None (alignment-group analysis)	Grouped by two-week postoperative alignment (Group A: residual exotropia vs. Group B: ≤10 PD esotropia vs. Group C: >10 PD esotropia)

Primary Outcome: Motor Alignment Success (≤10 PD)

Definitions were broadly aligned with the review threshold of ≤10 PD, although some studies allowed a small esodeviation of (≤5 PD) (Table 3). The pooled (weighted) surgical success rates across six studies were 73.7 ± 11.2%, with individual studies ranging from 48% to 90%. Primary endpoints ranged from 12 weeks to ≥2 years. When multiple follow-up points were reported, the longest available time point was extracted.

**Table 3:** Primary outcome: motor alignment success (≤10 PD). PD: prism dioptres; PR: plication-recession; R&R LR: recession and MR resection; AS: adjustable suture; NAS: non-adjustable suture; BTA-BLR: botulinum toxin augmented bilateral LR recession; BLR & uMR: bilateral LR recession and unilateral MR resection Grouping was based on early alignment, and success was assessed at the final follow-up as defined by each study.

Study	Success definition	Primary endpoint	Success
Ahn et al., 2018 [6]	Orthophoria to exodeviation ≤10 PD	≤2 years	Overall 69%; Group A 89%; Group B 57%
Lekskul et al., 2018 [10]	Exodeviation <10 PD	≥6 months	84.19%
Kimura et al., 2017 [11]	Esotropia ≤5 PD to exotropia ≤10 PD	12 months	PR 67%; RR 60%
Babu et al., 2018 [14]	Exodeviation <10 PD	3 months	AS 90%; NAS 85%
Mattout et al., 2022 [15]	Esodeviation <5 PD or exodeviation <10 PD	1 year	BTA-BLR 47.6%; BLR & uMR 66.7%
Narayan et al., 2023 [9]	Esodeviation ≤5 PD and exodeviation ≤10 PD	≥3 months	Overall 61%; Group A 12/26; Group B 41/53; Group C 4/14

In prognostic cohorts, Ahn et al. reported a two-year success of 69%, higher in those initially overcorrected at week 1 than those orthophoric to mild exotropic [6]. Narayan et al. reported overall success of 61%, highest with early overcorrection ≤10 PD esotropia 41/53 vs. residual exotropia 12/26 or >10 PD esotropia 4/14 [9]. In comparative studies, Kimura et al. reported similar 12-month success for PR and R&R [11], and Babu et al. reported high three-month success for AS and NAS [14]. In the large-angle cohort, Mattout et al. reported higher one-year success with BLR & uMR than BTA-BLR [15]. Lekskul et al. reported 84.19% achieving <10 PD exodeviation at ≥6 months using an intended undercorrection strategy [10].

Functional Outcomes: Binocularity/Stereoacuity and Postoperative Drift

Sensory outcomes were inconsistently reported (Table 4). Ahn et al. reported good stereoacuity in 57% (26/46) at two years, with better stereo outcomes in the less-overcorrected early alignment group [6]. Lekskul et al. reported binocular gain of 45.5% and improved binocularity of 42.9% [10]. Mattout et al. reported modest stereopsis improvement at one year [15].

**Table 4:** Functional outcomes: binocularity/stereoacuity and postoperative drift. PD: prism dioptres; FU: follow-up; NR: not recorded, PR: plication-recession; R&R: LR recession and MR resection; AS: adjustable suture; NAS: non-adjustable suture; BTA-BLR: botulinum toxin augmented bilateral lateral rectus recession; BLR & uMR: bilateral LR recession and unilateral MR resection Grouping was performed based on early alignment.

Study	Binocularity/stereoacuity (definition)	Binocularity/stereoacuity	Postoperative drift (timepoints)	Postoperative drift (PD)
Ahn et al., 2018 [6]	Good stereoacuity (≤100 arcsec)	Good stereoacuity 26/46 (57%); better stereo improvement in Group B	Week 1 to last FU	Group A 9.7 esodeviation ± 6.1 to 1.6 exodeviation ± 3.7 vs. Group B 2.0 exodeviation ± 2.7 to 6.8 exodeviation ± 5.6. Group A greater drift.
Lekskul et al., 2018 [10]	Binocular vision gain; improved binocularity	Gain 45.5% (92/202); improved binocularity 42.9%	NR	NR
Kimura et al., 2017 [11]	Post-operative stereoacuity not evaluated	NR	Week 1 to last FU	PR 6.8 ± 7.0 vs. R&R 10.7 ± 9.8. PR less drift.
Babu et al., 2018 [14]	Binocularity grade ≥2	Grade ≥ 2 (AS 9/20, NAS 15/20)	Day 1 to 3 months	Near AS 2.3 vs. NAS 2.2; distance AS 2.8 vs. NAS 2.8.
Mattout et al., 2022 [15]	Stereopsis improvement (≥2 octaves)	BTA-BLR 3/21 (14.3%) vs. BLR & uMR 5/30 (16.7%)	Week 1 to 1 year	BTA-BLR 25 esodeviation to 20 exodeviation; BLR & uMR 5 esodeviation to 6 exodeviation
Narayan et al., 2023 [9]	Not routinely carried out due to variability in response	NR	2 weeks to final FU	Group A 7 esodeviation ± 5 to 17.1 exodeviation ± 5, Group B 6 esodeviation ± 4 to 3 exodeviation ± 6, Group C 15 esodeviation ± 3 to 8 exodeviation ± 7

Where quantified, most studies demonstrated a progressive exotropic drift over time. Ahn et al. reported greater drift from week 1 to two years in the initially overcorrected group [6]. Kimura et al. reported a smaller total drift with PR than R&R [11]. Babu et al. reported a small, similar drift at three months [14]. Narayan et al. described progressive exotropic drift from two weeks to final follow-up [9]. Drift was not consistently quantifiable in all studies.

Safety: Diplopia, Adverse Events and Reoperation

Postoperative diplopia patterns differed by early alignment and use of botulinum toxin (Table 5). Ahn et al. observed earlier intermittent diplopia in the initially overcorrected group (Group A 8/18 vs. Group B 2/28), resolving by six months [6]. Lekskul et al. reported no postoperative diplopia [10]. Kimura et al. reported early diplopia more frequently after R&R than PR [11]. Mattout et al. reported higher early diplopia in BTA-BLR than BLR & uMR, resolving within one month; one patient developed mild ptosis following BTA that resolved within two months [15]. Narayan et al. reported diplopia in 4% during follow-up [9].

**Table 5:** Safety: diplopia, adverse events and reoperation. NR: not recorded; PD: prism dioptres; PR: plication-recession; R&R: LR recession and MR resection; BTA-BLR: botulinum toxin augmented bilateral LR recession; BLR & uMR: bilateral LR recession and unilateral MR resection; FU: follow-up Grouping was performed based on early alignment.

Study	Diplopia	Adverse events	Reoperation
Ahn et al., 2018 [6]	Intermittent diplopia early in Group A (8/18) vs. Group B (2/28); resolved ≤6 months	NR	NR
Lekskul et al., 2018 [10]	No postoperative diplopia reported	NR	Reoperation 5.13% (12/234) (all had postoperative exodeviation >15 PD; preoperative deviation ≥40 PD)
Kimura et al., 2017 [11]	Week 1: PR 9% (4/45); R&R 26% (11/43)	NR	Reoperation PR 7% (3/45); R&R 2% (1/43)
Babu et al., 2018 [14]	NR	Persistent red eye 5% (2/40)	NR
Mattout et al., 2022 [15]	BTA-BLR 42.9% (9/21) vs. BLR & uMR 16.7% (5/30); resolved within one month	Mild ptosis BTA-BLR 4.7% (1/21), resolved	NR
Narayan et al., 2023 [9]	4% in the FU period	NR	NR

Other adverse events were uncommon. Babu et al. reported two cases of persistent red eye, and Mattout et al. reported one case of mild ptosis following botulinum toxin, which resolved [14,15]. Reoperation reporting was inconsistent. Lekskul et al. reported 5.13% (12/234) undergoing reoperation, associated with larger postoperative residual deviations [10]. Kimura et al. reported reoperation within 12 months in 7% (3/45) of the PR group and 2% (1/43) of the R&R group [11]. Babu et al. reported no reoperations. Reoperation was not reported in several studies [14].

Discussion

Adult IXT remains under-represented in the strabismus literature, with most research focusing on paediatric cohorts. Adult IXT is challenging because adults have reduced functional reserves, are unable to suppress mild esodeviations causing diplopia, and may show exotropic drift over the long-term [16,17]. The expectations from adults are also beyond PD; they pay attention to stable alignment in real-life situations, such as driving, cosmetic appearance, and can directly impact their quality of life [18,19]. We therefore undertook this systematic review to synthesise evidence from current studies in evaluating surgical outcomes for adult IXT.

The studies of successful surgical outcomes were primarily based on the alignment criteria of ≤10 PD, with some studies allowing a small degree of esodeviation. Some studies have also evaluated post-operative stereoacuity status with different definitions of stereoacuity and binocular gain. The mean surgical success rates for all six studies were 73.7 ± 11.2%, ranging from 48% to 90%. Studies have reported multiple follow-up endpoints, which varied widely from 12 weeks to two years. Surgical success tends to decline over time; for example, Kimura et al. reported that success rates declined from 89% at week 1 to 55% at last follow-up in the PR group [11]. The reported success rate in Babu et al. is higher at 90%, as it defined surgical success at relatively shorter follow-up at three months [14]. Five studies have also documented progressive exotropic drift with time, with drift greater in either explicitly quantified as drift or evident from serial postoperative alignment measurements. In Narayan et al., serial measurements showed a consistent trend toward increased exodeviation [9]. The pattern of longitudinal alignment change is also seen in the large-angle cohort, particularly in the BTA-BLR group [15]. In the BTA-BLR group, surgical success was higher in smaller preoperative angles and good preoperative stereoacuity [15]. Babu et al. recorded that the mean drift was small and similar, consistent with limited drift being captured at short follow-up. These findings indicate that both early alignment state and procedure choice may influence long-term stability from progressive exotropic drift. As IXT typically exhibits exotropic drift over time, shorter follow-up tends to inflate apparent success when compared with longer-term assessment. This highlights the importance of standardised long-term endpoints.

A small and early esodeviation may predict better later success. In Ahn et al., patients who were initially overcorrected (within 10 PD esodeviation) marked higher success within two years (89%) than those who were orthotropic/mildly exotropic (57%), despite being greater drift in the overcorrected group. Similarly, Narayan et al. found best outcomes when overcorrection was within 10 PD of esodeviation compared with residual exotropia or larger esodeviation >10 PD [9]. These findings support the principle that early residual exotropia may be a prognostic factor in surgical success. The progression of exodrift could persist for up to three to four years after surgery; therefore, the strategy for overcorrection may seem like a good strategy to guarantee long-term alignment [20]. Residual exotropia has a higher rate of occurrence, and an overcorrection of <12 PD is associated with a more favourable outcome [21,22]. The trade-off in surgical dosing is the core challenge, as being conservative reduces the risk of diplopia but increases the risk of exotropic drift. A controlled and mild early overcorrection is vital, as excessive overcorrection performs poorly.

Binocular function can directly impact daily quality of life [18]. Binocularity and stereoacuity outcomes were inconsistently reported across the six studies, with only four providing any postoperative sensory assessment and with substantial heterogeneity in measurement and definitions. The definitions varied across Titmus stereoacuity thresholds, amblyopia categories, and study-specific gradings, which limit cross-study comparison or pooling. When reported, some adults demonstrated meaningful sensory improvement after surgery: Ahn et al. found good stereoacuity of ≤100 arcsec in 57% at two years, and Lekskul et al. reported binocular gain in 45.5% and improved binocularity in 42.9%, suggesting that restoration of alignment can be associated with improved binocular function in a subset of adults [6,10]. However, improvements were modest with the Mattout et al. cohort [15]. This may be due to the relatively larger angle IXT nature of this cohort and a plausible, stringent stereopsis threshold, e.g. ≥2-octave Titmus change instead of a single cut-off for good stereoacuity [15]. A large angle is more difficult to correct, as it reflects longer disease duration and decompensation, and therefore, patients have weaker fusional reserves to maintain alignment. Overall, the sensory outcomes are non-standardised with different sensory thresholds. They are an important, successful clinical marker, as motor alignment success may not directly translate into functional success.

The principal study methodologies varied across cohorts, with different group comparisons such as AS, different surgical techniques, use of botulinum toxin and uniform application of undercorrection strategy [23]. In Kimura et al., the total drift was smaller with PR than with R&R, suggesting potential advantages of PR [11]. Plication may strengthen the muscle without excision, potentially reducing tissue disruption and late remodelling compared with resection [24]. Moreover, other authors have also hypothesised that the initial tethering effect of resected muscle is weakened due to inflammation and adhesions between muscles [25,26]. However, given the retrospective design, differences may also reflect surgical dosing and selection bias. AS techniques allow surgeons to immediately alter ocular postoperative alignment and prevent overcorrection or undercorrection. Nevertheless, Babu et al. demonstrated that there were no clear advantages in using AS compared to NAS in achieving a successful surgical alignment [14]. This finding, however, is based on a small sample size with a short-term follow-up of three months.

In a large angle IXT cohort, BTA-BLR demonstrated a higher rate of undercorrection from the early postoperative period compared to three-muscle surgery (uMR & BLR) [15]. This pattern suggests that a two-muscle weakening strategy with botulinum toxin may provide an insufficiently effective surgical dose when compared with a permanent correction of a three-muscle approach. This also reinforces that the higher undercorrection rate is unlikely to solely be explained by the waning effect of botulinum toxin A with time [27]. The lower predictability of botulinum toxin has also been acknowledged in their cohort [15]. In applying a uniform and intentional undercorrection of the 5 PD strategy, the success rate is considerably higher at 84% compared to most cohorts [10]. The rationale for using an undercorrection strategy is that adults with poor functional reserve typically experience persistent postoperative diplopia from overcorrection [10,28]. There were also no cases of consecutive esotropia, and only a small proportion of patients required reoperation. This observation brought an insight into current surgical practice that a careful dosing and undercorrection strategy may help improve surgical outcomes.

Safety reporting was inconsistent across the six studies, with postoperative diplopia being the most frequently described adverse outcomes and other complications were rarely documented. Overall, the diplopia rates were low, but with varied definitions, duration and management strategies were variably reported. Diplopia appeared to relate to early postoperative alignment and BTA. Intermittent diplopia occurred more commonly in the initially overcorrected group despite having a higher success rate, but it resolved within six months [6]. There was no postoperative diplopia reported in the undercorrection strategy for avoiding symptomatic overcorrection in adults. In Kimura et al., diplopia at postoperative week 1 was more frequent after R&R than PR (26% vs. 9%), suggesting technique or early alignment differences may influence early symptoms [11]. In the large-angle cohort, Mattout et al. reported higher early diplopia in the BTA-BLR group (42.9%) than the uMR & BLR group (16.7%), with symptoms resolving within one month [15]. Narayan et al. reported diplopia in 4% during follow-up, although stereopsis testing was not routinely performed [9]. Across cohorts, diplopia rates were low and resolved within a few months. Adverse events unrelated to diplopia were sparsely reported. There were some cases of mild ptosis in the BTA-BLR cohort, and persistent red eye in patients [14,15]. Beyond these, complications such as infection, slipped/lost muscle, significant motility limitation or persistent consecutive esotropia were not consistently described, limiting conclusions about the true complication profile.

Reoperation reporting was also inconsistent. Lekskul et al. reported a reoperation rate of 5.13% (12/234), associated with larger residual postoperative deviations, while Kimura et al. reported reoperation within 12 months in 7% (3/45) of the PR group and 2% (1/43) of the R&R group [10,11]. Babu et al. reported no reoperations, but follow-up was short, and outcomes were defined early [14]. Reoperation was required in these patient cohorts due to persistent large exodeviation (>15 PD) and exotropic drift. There were no other indications for reoperation, such as slipped or lost muscle.

Future research in adult IXT should prioritise comparing common surgical strategies and technique variations, using standardised success definitions and fixed long-term follow-up endpoints. Given the consistent observation of exotropic drift, studies should pre-specify and report a drift metric alongside motor alignment. Outcomes should extend beyond PD to include diplopia burden, control, binocular function and validated patient-reported quality-of-life measures. Large-angle adult IXT requires targeted evaluation of optimal surgical dosing and the role of botulinum augmentation.

Limitations

This review has several limitations. First, study quality was variable; five of six included studies were retrospective, increasing susceptibility to selection bias, confounding and incomplete reporting. Second, there was substantial clinical and methodological heterogeneity across studies, including differences in baseline deviation severity procedures performed, age ranges, botulinum augmentation and follow-up duration, which precluded meta-analysis and limited direct comparability of success rates. Third, although most studies defined motor success using a ≤10 PD exotropia threshold, several allowed small esodeviations, introducing threshold heterogeneity. Fourth, functional outcomes were under-reported and inconsistently defined: only a subset of studies evaluated stereopsis, using different instruments and thresholds and quality of life outcomes were not routinely captured. Fifth, safety reporting was inconsistent, particularly for persistent postoperative diplopia and reoperation thresholds, which limits confidence in true adverse events. Finally, associations between early overcorrection and later success should be viewed as observations and not causal inference, because early postoperative alignment groups were not randomised in retrospective studies.

## Conclusions

In adults undergoing surgery for IXT, the mean successful alignment rate was 73.7%. Outcomes varied widely with baseline preoperative deviation, surgical approach, and follow-up duration. Where reported, most studies demonstrated progressive exotropic drift over time, underscoring the importance of long-term assessment. Evidence from alignment-stratified cohorts suggests that early postoperative alignment is prognostic, with small early overcorrection associated with higher later success than early residual exotropia, although excessive overcorrection performs poorly and may increase diplopia risk. In the included large-angle cohort, three-muscle surgery achieved higher one-year success than botulinum-augmented BLR within the same study, suggesting very large deviations may require a greater and more predictable permanent surgical dose. Functional outcomes and safety endpoints were inconsistently reported, highlighting the need for prospective adult-only studies with standardised definitions, fixed follow-up endpoints, drift metrics, and patient-centred outcomes.
